# Hygiene, Sanitation, and Water: What Needs to Be Done?

**DOI:** 10.1371/journal.pmed.1000365

**Published:** 2010-11-16

**Authors:** Sandy Cairncross, Jamie Bartram, Oliver Cumming, Clarissa Brocklehurst

**Affiliations:** 1 London School of Hygiene & Tropical Medicine, London, United Kingom; 2 Water Institute, Gillings School of Global Public Health, University of North Carolina at Chapel Hill, North Carolina, United States of America; 3 WaterAid, London, United Kingom; 4 UNICEF, New York, New York, United States of America

## Abstract

In the final article in a four-part *PLoS Medicine* series on water and sanitation, Sandy Cairncross and colleagues outline what needs to be done to make significant progress in providing more and better hygiene, sanitation, and water for all.

**Summary Points**

- As the last article in a series on water and sanitation, this paper considers what needs to be done to make significant progress towards ensuring universal access to hygiene, sanitation, and water.
- We first discuss the differences between these three subsectors and the possible reasons for poor rates of progress towards achieving universal access in recent years.
- Then, we consider the actors whose engagement is essential for the sector, including the poor households themselves who are significant investors, local and central government, donors, and international agencies.
- Finally, we discuss the potentially important role of the health sector in improving hygiene, sanitation, and water worldwide and propose a detailed Agenda for Action.

**This is one article in a four-part** ***PLoS Medicine*** **series on water and sanitation.**

## Introduction

The previous papers in this series have set out the importance for health of sanitation and water and touched on the importance of hygiene [1],[2],[3]. Three clear messages have emerged:

1. Unimproved hygiene, inadequate sanitation, and insufficient and unsafe drinking water account for 7% of the total disease burden and 19% of child mortality worldwide [4].
2. Interventions in hygiene, sanitation, and water are highly cost-effective and capable of preventing a large part of this devastating disease burden.
3. Progress in ensuring access to these basic services has been painfully slow in much of the developing world.

These three messages present an imperative for everyone concerned with improving health. The centrality of these issues to health has been made clear in numerous international declarations, but priority and progress remain inadequate. As it stands, the world will not deliver the Millennium Development Goal (MDG) targets on water in many poor countries and on sanitation in most, let alone achieve the vision of universal access.

This paper analyses the causes of poor national progress, discusses how these can be addressed, and highlights the potential roles of the various actors—especially the health sector—in tackling the challenges that lie ahead.

## Hygiene, Sanitation, and Water – One Sector or Three?

Traditionally, sanitation and water, together with hygiene, have been treated as a single sector, but the examination of this sector’s component parts in this series has revealed not only that they have much in common but also that much sets them apart.

Common to the three subsectors is the extent to which they impact upon mortality and morbidity burdens in the developing world. There has been some debate about whether the respective health benefits of hygiene, sanitation, and water supply are additive. A literature review two decades ago [5] found the reduction of diarrhoea in studies involving two or all three interventions was no greater than in studies involving only one. It would be unwise to draw any firm conclusion from this finding because of the small number of studies included in this review, the wide range of settings, and the variable epidemiological rigour of the studies. Moreover, a more recent systematic review [6] reported that hand washing with soap has a similar impact on diarrhoea in industrialised and developing countries, where water supply and sanitation differ greatly, and in a study in Brazil the impact of sanitation on diarrhoea was not affected by the high level of on-plot water supply coverage [7]. Results such as these suggest that the impact of each subsector can be treated independently.

Separate or together, the three components are critical determinants of health. Achieving universal access to safe drinking water, adequate sanitation, and improved hygiene, and progressively improving the level and quality of services are essential steps on every country’s journey to securing good health for its citizens.

However, it is not usually practical to integrate water supply with sanitation and hygiene promotion, even though they are all parts of an environmental strategy to prevent faeco-oral infections. Hygiene promotion and sanitation promotion both suffer from the budgetary dominance of water supply, and from a loss of effectiveness when implemented too fast [8]. Freeing sanitation promotion from its link with construction (of toilets or of water supplies) avoids these problems and makes it more suitable for implementation by the health sector. Indeed, some of the most successful sanitation programmes in the developing world, such as the rural sanitation programmes of Ethiopia and Benin (see Box 1 in the Sanitation and Health paper of this series [3]) or some of the recent Community-Led Total Sanitation programmes [9], have been implemented by the health sector.

**Box 1. Agenda for Action in Seven Domains**

**Note:** In this box, “hygiene, sanitation, and water” is abbreviated to “HSW.”

**1. HSW in health policy – and vice-versa**

*All to:*

- recognise HSW as one of the key intervention strategies for reducing morbidity, mortality, and health care costs.
- commit to working across sectors to achieve better results for health.

*WHO to:*

- call on health ministries and international health agencies to strengthen intersectoral policy and to build effective multisectoral coordination mechanisms on HSW and health.

*National governments to:*

- emphasise HSW within national development plans, such as Poverty Reduction Strategy Papers, as a health priority.
- ensure that Finance and Planning Ministries are aware of the evidence and impacts of low levels of HSW coverage.
- ensure that every child has access to HSW in school and that no new schools are constructed without HSW facilities.

*National health ministries to:*

- include HSW as an essential component of all health and child health policies and plans with an adequate and costed strategy.
- include targets and plans for the achievement of universal HSW coverage alongside other universal health coverage targets.
- include HSW as a key performance indicator of management in the health sector.
- develop criteria for more equitable allocation of resources to ensure better focus on serving the unserved.

*Local government to:*

- work with all local partners (civil society and private service providers) to coordinate plans for universal HSW coverage for better health.
- ensure that local health and development strategies and plans include HSW.

*Major donors to:*

- include HSW in national assistance strategies for the health sector.
- target resources better to the unserved with the aim to ensure at least 50% of aid for water and sanitation goes to low-income countries and 27% to basic services.

**2. HSW in health institutions**

*WHO to:*

- establish international benchmarks for HSW-related needs in health care settings.

*Health Ministries to:*

- ensure a statutory requirement that all health care facilities have adequate and safe HSW.
- monitor coverage and maintenance of HSW in health care facilities.

*Health care facility managers to:*

- take responsibility for ensuring access to and use of HSW by all staff.

*All health care workers to:*

- practise appropriate hygiene in day to day work to provide a model of good practice to patients and visitors.

**3. HSW in health research**

*WHO to:*

- convene a multi-agency, stakeholder conference of research funders, providers, and users to define the research needs in health aspects of HSW.

*Research funding agencies and donor governments to:*

- consider how they can improve their support for critical research on HSW and health, and for operational and formative research as a part of normal HSW programmes.
- build capacity for research in HSW in those countries where HSW coverage is low and the related disease burden is high.
- invest in the development of national leaders who can champion this research agenda and contribute to tackling the HSW challenges in their own countries.
- ensure assessment of the quality and impact of the outputs of research based in developing countries, taking full account of the impact of that research in developing countries.
- invest in HSW research beyond health, especially in the area of sustained behaviour change, economic and social impacts, sustainability, technology, and in measures to ensure the dissemination and application of the findings.

*Developing country governments to:*

- identify and invest in potential research leaders amongst their own scientists and academics capable of taking forward research relevant to local communities’ needs.

**4. HSW in health surveillance**

*WHO and other UN agencies to:*

- review progress against the MDG target on sanitation and water as a “health MDG” at the World Health Assembly
- develop guidance on surveillance of HSW-related diseases.
- strengthen HSW Health indicators in Demographic Health Surveys (DHS) and Multiple Indicator Cluster Surveys (MICS) and cross reference these with disease burdens and trends.
- develop and propagate improved and sensitive indicators of environmental health to include HSW.

*Health ministries to:*

- evaluate and improve their systems for the surveillance of diarrhoeal disease in children and of other diseases linked to inadequate HSW.
- ensure Health Management Information Systems (HMIS) to include direct or proxy coverage indicators for HSW.

*Major donors to:*

- adequately audit their assistance to assess progress in delivering HSW-related health outcomes via the health and other sectors.
- facilitate cross-sector financing for health and HSW to secure more rapid progress on health outcomes.

**5. HSW in health delivery programmes**

*National governments to:*

- review roles and responsibilities across sectors for accelerated progress towards universal HSW coverage.
- develop national health strategies for the reduction of diseases linked to inadequate HSW, and to ensure that these are implemented and adequately funded.

*Health ministries and local government to:*

- ensure that health delivery programmes adequately address HSW.
- review health training on HSW at all levels—and in particular for health extension workers or national equivalent.
- brief all health personnel at all levels on roles and responsibilities relating to HSW.
- develop local capacity for implementation of HSW programmes, in both public and private sectors.

**6. HSW in regulations and standards to protect health**

*WHO to:*

- continue to develop their guidelines with increased emphasis on assisting and advising countries in adapting them to national needs and into national legislation.

*Developing country governments to:*

- revisit public health legislation and enforcement procedures.

*Health Ministries to:*

- review the adequacy of and, where appropriate, strengthen regulations and standards and their implementation aimed at reducing disease by enhancing the quality and extending coverage of HSW.
- carry out environmental health impact appraisal of proposed legislation, and publish the potential impacts on public health of all legislation that may positively or negatively impact on HSW.

*Local government to:*

- develop and apply building codes and bylaws that are instructive and supportive to those seeking to install affordable sanitation technologies, and ensure that landlords fulfil their obligation to provide housing with adequate sanitation for their tenants.

**7. HSW in health advocacy**

*WHO and other United Nations Organizations to:*

- lobby donors to ensure that HSW are reflected in their health department agendas as well as their infrastructure agendas, and to direct more of their aid budget to HSW.
- continue the initiative of the JMP and the GLAAS report [12] in holding countries and donors to account for their contributions to the progress of HSW coverage.

*Major donors to:*

- engage aid recipient governments in dialogue to strengthen national health strategies and plans to deliver universal HSW coverage.

*Health and public health professionals to:*

- inform developing country and international policy-makers of HSW-related disease burden.
- call on their governments to provide leadership and allocate adequate resources towards universal HSW coverage.

*Nongovernmental organisations from the health and HSW sectors to:*

- support a call for universal access to HSW for all.
- call on governments in the developed and developing world to take action urgently to address this health issue.

## Sectoral Stagnation

### Political Neglect

The previous papers in this series give examples of poor performance in delivering progress on sanitation and water. This inadequate performance is not inevitable; some extremely poor countries have provided water supplies to half their population (for example, Burkina Faso, Ghana, and Guatemala), or doubled their sanitation coverage (for example, Benin, Ethiopia, and Niger), since 1990 [10]. Where performance is poor, it is rooted in a lack of political will, which is evidenced by the low priority afforded to water and sanitation in government and donor policies and budgets.

### Low Level of Ambition

Political neglect is compounded by a low level of ambition. The 1978 Alma Ata Declaration on Primary Health Care was clear in its call for action by “all governments, all health and development workers, and the world community to protect and promote the health of all the people of the world.” This vision of universal access to health included a specific call for, at least, “an adequate supply of safe water and basic sanitation” [11].

However, the MDG target for water and sanitation aims only to reduce by half those without access to these services between 1990 and 2015. For hygiene, there is no target. Even if the MDG targets are met, a quarter of the world’s population will still be without access to even a basic toilet and one in ten will be without access to an improved water source. Sadly, many countries will not meet even these modest targets. The international development community will shortly enter a phase of review, revision, and perhaps recrimination on the rate of progress toward the MDGs. Further goals are then likely to be adopted for the period after 2015 and it is hard to see how these could be less than universal access to water and sanitation at the home, health centre, school and workplace. However, these new goals must give priority to the most disadvantaged and encourage progressive improvement of service levels.

### Poor Performance

The WHO/UNICEF Joint Monitoring Programme (JMP) recently reported that the world is on track to meet the MDG target for water but is seriously off-track for sanitation [10]. Closer examination of the report paints a bleak picture for the world’s poorest countries and regions. Africa, at current rates of progress, will not meet the MDG target for water until 2035, or the sanitation target until 2108. Failings at all levels lie behind this poor progress.

At the international level, water and sanitation are a low priority compared to other sectors such as health and education. Many donors, such as the UK and Norway, give water and sanitation just 1.5% of their total development budget. Moreover, aid for the sector is poorly targeted with only 24% of it going to the Least Developed Countries between 2002 and 2006, and there is no relationship between allocation of aid and the level of access to water and sanitation in a given country [12]. In short, aid for the sector is not getting to where it is most badly needed.

At the national level, policy and plans are weak or absent, and effective action is undermined by institutional fragmentation and poor coordination within and outside government. Allocations within national budgets—particularly for sanitation—are low and largely financed by aid rather than by national revenue. In Zambia, for example, in 2008, 91% of the government allocation for sanitation was from external aid [13].

At the local government level, responsibility for delivering these services has been decentralised without the necessary financing or requisite investment in local capacity. In addition, local resources for the sector are often off-budget for local government, leading to little in the way of capital budget for expanding infrastructure and poor targeting of investments [13].

Several features of the development context further complicate progress towards the coverage goal. Rapid population growth, especially in towns and cities, makes it necessary for sector coverage to run in order to stand still. Climate change, with diminishing rainfall in relatively dry regions and increasing seasonality of river flows, is also hindering progress towards the MDG targets.

## Building the Systems to Deliver

The challenge of meeting the MDG target for water and sanitation by 2015 and, beyond that, realising a vision of universal access, is immense. The funding requirement is not the least of the obstacles to meeting this challenge. Estimates of the global cost of meeting the MDG target range from US$6.7 billion to US$75 billion per year [12]. Yet, the global total in 2008 of aid disbursements for sanitation and water supply by OECD members and several multilateral agencies was only US$5.3 billion [12]. Furthermore, most of these estimates do not include the costs of support services or institutional capacity to plan, build, and manage the infrastructure.

It would be simplistic, however, to blame a lack of funding for all the poor progress in the sector, particularly since numerous well-known instances in which reduced subsidy has led to improved performance suggest that there is not a linear relationship between money and progress.

Much of the debate on how to meet the challenge of the MDG target has focused on how successful community or neighbourhood projects can be “scaled up.” There are many examples of highly successful local innovations in water supply, but few have scaled up beyond the district level. In sanitation, so few projects have achieved the construction of more than, say, 10,000 units that the same exceptions are endlessly cited in the literature (e.g., [14]). For hygiene promotion, there appears to be a trade-off between quality and scale, with a tendency for effective and participatory local projects to degenerate into hectoring didacticism when scaled up.

This focus on “scaling up”—if it can work at a small scale, how can it be made to work at a large scale?—can detract from the root causes of poor progress at the national level. Rather than an absence of small-scale success, the major challenge lies in the weak and often under-resourced public institutions mandated to deliver these services or oversee their delivery. The challenge in effect is not how successful pilots can be “scaled up” but how progress can be delivered “at scale.”

## The Key Actors and Their Roles

### Households

Individual households have largely been viewed by government technicians as passive recipients of water and sanitation services, ignoring the extent to which many have provided their own services. Of the nine million hand pumps in Bangladesh, for example, two-thirds are privately owned and maintained [15]. So are most of the world’s pit latrines. For example, Kampala’s population grew by 600,000 from 1992 to 2003 and in 2003 about half of the additional population were using their own pit latrines whereas there had been negligible growth in the use of shared latrines provided by landlords or for public use [16].

Even the poor aspire to become formal customers of local utilities, with the rights and privileges that entails. They are often willing to pay much higher tariffs than service managers expect, having previously paid larger sums (for poorer service) to providers in the informal sector [17]. The facilitation of access to water and sanitation services by poor households will enable them to exert their rights as customers and to press for service improvements by consumer demand as well as political, legal, or other means.

If households in rural and periurban areas are encouraged to invest in their own wells or other water and sanitation facilities, they are likely to choose cheaper, simpler technologies and to help to maintain them. Government technicians may consider these technologies inferior, but the affordability and feasibility of construction and maintenance by local people are advantages of such self-supply arrangements. Using scarce external resources to give relatively high-quality services to a few of the unserved raises equity issues; it is preferable to spread the funds around and have them work harder for more people. It has been estimated [18] that some 25 million people in sub-Saharan Africa could improve their own water supplies through the use of affordable technology. Public sector and external funding can help to develop such technology and bring it to market. For instance, UNICEF is developing the market for low-cost manual drilling of tube wells by artisans in West Africa.

Already, most of the investment in water supply and sanitation comes from households, as illustrated above. Donor investments are scarce, and, although government allocations are greater, they are insufficient to “buy” enough coverage to meet the MDGs, much less provide universal access. A key challenge is thus to use the available funding most effectively, with a mix of catalytic government and donor interventions, and wisely stewarded household investments.

### Local Government

Effective delivery of water and sanitation services is usually best done at a local level. The strength and accountability of local government will therefore be a key determinant of the coverage and sustainability of those services, both in villages and in more urban settings.

For rural community water supplies, the key problem is the responsiveness of local government, including village institutions, to their maintenance needs. Rural water systems may break down for technical reasons, but when they are not mended promptly, the reasons are primarily institutional, not technical [19].

In sanitation, the challenge for local government is to work with the existing providers (usually artisan latrine-builders who may have spent their lives avoiding government interference) on product development, demand stimulation, marketing, quality assurance, and co-ordination of the final disposal of wastes [16].

### Central Government

Better outcomes in hygiene, sanitation, and water will only be obtained from local government if it is supported by central government. This support can take the form of resourcing and regulation.

*Resourcing* refers in the first instance to financial resources. Local municipalities and district councils cannot be expected to be enterprising if they live from hand to mouth. The current trend to decentralisation offers a possibility, but no guarantee, of increased distribution of central resources in the future [12].

Funding can also be used to redress disparities or as an incentive for action. For example, in Myanmar in the 1980s, any district seeking funding for rural sanitation was required to submit a detailed plan of action endorsed by all relevant local officials to the Health Ministry and had to offer its own counterpart contribution (U. Myint, personal communication). This demand-responsive approach has also been used more recently in rural water supply and sanitation programming in India, Sri Lanka, Ghana, and other countries. This approach, although not always well-implemented, illustrates how central government or donor funds can be used strategically.

*Regulation* is better understood in the context of privatised water companies, but central government often uses its powers to control the actions of local authorities and its legislative action can also empower local authorities to enforce bylaws. It is no coincidence that in England, the 1872 Public Health Act came only one year after the 1871 Local Government Act, which reformed the local government system and gave it the moral and legal authority to enforce the new public health regulations [20].

Bylaws can play a hugely important role in promoting sanitation. Some low-cost sanitation schemes in cities have been impeded (or even abandoned) by the blind application of outdated building regulations that make some aspect of the technology illegal or that impose technology standards that are simply too expensive such as Senegal’s periurban standard $500 latrine with two pits for alternate use. More positively, in Bobo Dioulasso (Burkina Faso) and in some villages in Mozambique during the early years of the country’s independence, the construction of a toilet was made a condition of ownership of each residential plot of land.

### External Support Agencies

External support agencies, like governments, need to invest more in hygiene, sanitation, and water but at the same time need to use their resources more strategically to leverage the investments of households, local communities, and government bodies.

In line with the Paris declaration on aid effectiveness [21], the bilateral donor agencies are committed to moving their aid from project grants to budgetary support, either for a sector-wide approach (SWAP) or in a common fund to implement a multisectoral Poverty Reduction Strategic Plan (PRSP). In either case, funding is provided on the agreement that the recipient government follow specified principles in spending it. We would like to see some of the approaches to improving sanitation and water supply proposed in this Series embodied as principles in SWAP agreements and PRSPs. We would also like to see an end to donors and NGOs pressuring governments to apply or increase hardware subsidies in sanitation programmes in the belief that they will increase uptake rates despite the well-documented corrosive effects of this approach [22],[23].

## Roles and Responsibilities of the Health Sector

The health system does not have the vocation or the resources to take over the construction of water and sanitation works, or other tasks in the sector, which are normally managed by engineers. But improved hygiene, adequate sanitation, and safe drinking water are cost-effective, life-saving interventions critical to securing progress on the health MDGs and reducing the global disease burden. They are central within the “health system” as defined by WHO [24]:

“A health system consists of all organizations, people and actions whose primary intent is to promote, restore or maintain health. This includes efforts to influence determinants of health as well as more direct health-improving activities. … It includes inter-sectoral action by health staff … .”

How can the health system be comprehensively strengthened, not just to provide health care but also to ensure that progress on improving health is not undermined by poor progress on hygiene, sanitation, and water? The first paper in this series [1] listed six roles for the health sector in accelerating progress on hygiene, sanitation, and water [25]. Here, we focus on the three roles with an intersectoral dimension, namely: advocacy (amplifying the importance of hygiene, sanitation, and water in intersectoral dialogue); regulation (ensuring adequate quality of service); and promotion (stimulating household and community action).

### Advocacy

There are various possible dimensions for the advocacy of health professionals regarding hygiene, water, and sanitation in the intersectoral arena. Here, we list these dimensions and give an historical example of each.

- *Advocating for adequate resources on the basis of health data*

The historical record for England shows the important role of medical pioneers such as William Farr at the General Registry Office in collecting and publishing data, documenting the environmental health risks of 19th century urban life, identifying sewage contamination of water supplies as the main cause of cholera epidemics, and creating conditions for competition between cities to achieve the lowest infant mortality rate [20].

- *Formulating comprehensive national health strategies that include environmental health*

In early 20th century England, a high infant mortality rate was among the criteria by which local authorities were judged eligible by the Local Government Board for loans to build water supplies [26].

- *Leading intersectoral dialogue on hygiene, sanitation, and water as health interventions in communities, homes, clinics, and schools*

W. N. Pickles, a country doctor in Yorkshire in the 1930s, documented an epidemic of dysentery spread by contaminated towels in a school toilet. He used his findings to advocate school hygiene improvements, on the grounds that “Knowledge is not the only thing which is disseminated in these institutions” [27].

In developing countries today, it is a public health scandal that many schools and health facilities are built without water supplies or toilets, or that these facilities are not adequately maintained. A survey of 42 developing countries in Africa and Asia found that only one in four could give the rate of coverage of primary schools with sanitation. More worryingly, half of the countries that did provide this information reported that fewer than 50% of their rural primary schools have sanitation [12]. Lack of data on water and sanitation in schools is a serious constraint for advocacy and planning efforts. Out of 60 priority countries for UNICEF hygiene, sanitation, and water interventions in schools, only 27 have a national plan of action for those interventions.

In England a century ago, key roles were played by the Medical Officer of Health in each town, and by the Medical Department in the Local Government Board [28]. Similarly, in most developing countries today, the advocacy effort needs to be led by national and regional Directors in the health sector. Epidemiological data and data on the coverage and reliability of water and sanitation services are needed to aid this advocacy effort. In addition, selection and monitoring of indicators of exposure to health risks should provide an evidence base for more effective and equitable interventions, programmes, and policies [29].

Finally, a priority of the health sector’s advocacy effort should be to extend coverage and facilitate access to water by the poor. Where availability at standpipes is already good and water resources allow, a further objective is to advocate an increase the number of house connections as these are associated with a 63% reduction in diarrhoea when compared with a safe public water source [30]. Replacement of lump sum connection charges by a more affordable charge on the monthly tariff would help to achieve this.

### Regulation

In most countries, the regulatory roles of the health sector that are relevant to water and sanitation relate to drinking water quality and to building standards, respectively. With regard to the first, health officials should seek to extend their mandate to cover the quality of service (coverage, quantity, continuity, and cost) in addition to water quality [31]. With regard to the second, bylaws to ensure that sanitation facilities are constructed or available in the vicinity of new houses can be a powerful means to improve access to sanitation.

### Promotion

Outreach or health extension workers are found in many communities, especially those covered by Integrated Management of Childhood Illness (IMCI). In rural areas, this cadre is often mandated to promote hygiene and sanitation. Currently, though, community health is often neglected due to resource constraints, which result in many health workers being confined to clinics and health centres [32]. When such community-based health staff are told to give priority to hygiene and sanitation and are adequately supported, the results can be remarkable (see the Ethiopian example in the Sanitation and Health paper in this series [3]). Field workers in other sectors—agricultural extension officers, social workers, and so on—can also be mobilised to promote water, sanitation, and health as well as for their own sector if provided with the modest funds they need to visit their parish [33].

Promotion here includes activity to stimulate demand for sanitation, and also to effect changes in hygiene-related behaviour such as hand washing with soap. It is a function for which the health sector already has a vocation. Its many staff are in day-to-day contact with the public, and often have years of experience of behaviour change interventions. Their promotional voice is often amplified by volunteer community health workers [34] and expert patients who are able to provide highly effective peer education while distributing items such as medication and condoms. For hygiene and sanitation promotion, they are largely an unexploited resource.

## Conclusion

One of the greatest indictments of our age is that, despite knowing the cause, having the technology, and being able to mobilise the means to eliminate the problem, so many children in the world continue to die each year from easily preventable diseases that the developed world seems to have long forgotten.

The health sector has a crucial stake in remedying this situation by ensuring that hygiene, sanitation, and water receive the attention they deserve, and a clear role in addressing the challenge as illustrated in our proposed Agenda for Action (Box 1). Although involvement in hygiene, sanitation, and water may seem like an added burden for an overburdened, under-resourced health system, we prefer to see it as an opportunity to form alliances with other sectors, agencies, and communities, and to ensure that their resources are deployed not only to serve their objectives, but also in the service of public health. The time to grasp this opportunity is now.

## Abbreviations

IMCI: Integrated Management of Childhood Illness
JMP: WHO/UNICEF Joint Monitoring Programme
MDG: Millennium Development Goal
PRSP: Poverty Reduction Strategic Plan
SWAP: sector-wide approach

## References

[1] Bartram J, Cairncross S (2010). Hygiene, Sanitation and Water: Forgotten Foundations of Health.. PLoS Med.

[2] Hunter P, MacDonald AM, Carter RC (2010). Water Supply and Health.. PLoS Med.

[3] Lane J, Trouba D, Mara DD, Scott BE (2010). Sanitation and Health.. PLoS Med.

[4] Prüss-Üstün A, Bos R, Gore F, Bartram J (2008). Safer water, better health: costs, benefits and sustainability of interventions to protect and promote health.

[5] Esrey SA, Potash JB, Roberts L, Shiff C (1991). Effects of improved water supply and sanitation on ascariasis, diarrhoea, dracunculiasis, hookworm infection, schistosomiasis, and trachoma.. Bull WHO.

[6] Curtis V, Cairncross S (2003). Effect of washing hands with soap on diarrhoea risk in the community: a systematic review.. Lancet Infect Dis.

[7] Barreto ML, Genser B, Strina A, Teixeira MG, Assis AM (2007). Effect of city-wide sanitation programme on reduction in rate of childhood diarrhoea in northeast Brazil: assessment by two cohort studies.. Lancet.

[8] Cairncross S (1989). Water supply and sanitation: an agenda for research.. J Trop Med Hyg.

[9] Chambers R (2009). Going to Scale with Community-led Total Sanitation: Reflections on Experience, Issues and Ways Forward. IDS Practice Paper.

[10] UN-Water (2010). Progress on sanitation and drinking-water; 2010 update.

[11] WHO (1978). Declaration of Alma-Ata. International Conference on Primary Health Care, Alma-Ata, USSR, 6–12 September 1978.

[12] UN-Water (2008). Global Annual Assessment of Sanitation and Drinking-Water. Targeting resources for better results.

[13] WaterAid (2008). Think Local, Act Local: Effective financing of local governments to provide water and sanitation services.

[14] Anon (1995). The Orangi Pilot Project.. Environment and Urbanization.

[15] Caldwell BK, Caldwell JC, Mitra SN, Smith W (2003). Searching for an optimum solution to the Bangladesh arsenic crisis.. Soc Sci Med.

[16] Cairncross S (2004). The Case for Marketing Sanitation. Water & Sanitation Program Field Note.

[17] Cairncross S, Kinnear J (1992). Elasticity of demand for water in Khartoum, Sudan.. Soc Sci Med.

[18] Sutton S (2004). Preliminary desk study of potential for self supply in sub-Saharan Africa.. http://www.rwsn.ch.

[19] Davis J, Lukacs H, Jeuland M, Alvestegui A, Soto B (2008). Sustaining the benefits of rural water supply investments: Experience from Cochabamba and Chuquisaca, Bolivia.. Water Resour Res.

[20] Szreter S (1988). The importance of social intervention in Britain’s mortality decline c. 1850–1914: a re-interpretation of the role of public health.. J Soc Hist Med.

[21] Anon. (2005). Paris Declaration on Aid Effectiveness, Ownership, Harmonisation, Alignment, Results and Mutual Accountability. High level Forum, Paris, 28 Feb to 2 Mar 2005.

[22] Jenkins MW, Sugden S (2006). Rethinking Sanitation: Lessons and Innovation for Sustainability and Success in the New Millennium. Human Development Report, Occasional Paper.

[23] Kar K (2003). Subsidy or self-respect? Participatory Total Community Sanitation in Bangladesh. IDS Working Paper 184.

[24] WHO (2008). Everybody's Business: Strengthening health systems to improve health outcomes: WHO's framework for action.

[25] Rehfuess EA, Bruce N, Bartram JK (2009). More health for your buck; health sector functions to secure environmental health.. Bull WHO.

[26] Benjamin B (1964). The Urban Background to Public Health Changes in England and Wales, 1900–50.. Popul Stud.

[27] Pickles WN (1972). Epidemiology in Country Practice.

[28] Chave SPW (1980). The rise and fall of the medical officer of health.. J Public Health Med.

[29] Ezzati M, Utzinger J, Cairncross S, Cohen AJ, Singer BH (2005). Environmental risks in the developing world: exposure indicators for evaluating programs, interventions and policies.. J Epidemiol Community Health.

[30] Cairncross S, Valdmanis V, Jamison DT, Breman JG, Measham AR (2006). Water supply, sanitation and hygiene promotion.. Disease Control Priorities in Developing Countries (2^nd^ edition).

[31] Lin C (2005). Service quality and prospects for benchmarking: Evidence from the Peru water sector.. Utilities Policy.

[32] DFID, UNICEF, USAID, WHO (2003). The Analytic Review of the IMCI Strategy; Final Report.

[33] Cairncross S, Cutts FT, Periès H (1997). Vertical programmes; what are they good for?. Lancet.

[34] Gilson L, Walt G, Heggenhougen K, Owuor-Omondi L, Perera M (1989). National community health worker programs: how can they be strengthened?. J Public Health Policy.

